# A Rising Hope of an Artificial Heart: Left Ventricular Assisted Device - Outcome, Convenience, and Quality of Life

**DOI:** 10.7759/cureus.5617

**Published:** 2019-09-10

**Authors:** Suyeewin Thiha, Abdul Rehman Z Zaidi, Chris A Robert, Mohammed K Abbas, Bilal Haider Malik

**Affiliations:** 1 Internal Medicine, California Institute of Behavioural Neurosciences and Psychology, Fairfield, USA; 2 Internal Medicine, King Fahad Medical City, Riyadh, SAU; 3 Obstetrics & Gynecology, Sunrise Hospital, Pune, IND; 4 Internal Medicine, California Instititute of Behavioral Neurosciences and Psychology, Fairfield, USA; 5 Internal Medicine, California Institute of Behavioral Neurosciences and Psychology, Fairfield, USA

**Keywords:** lvad, heart failure, ejection fraction, left ventricular assist device (lvad), cost-effectiveness, quality of life

## Abstract

With the introduction of mechanical circulatory support, mainly continuous-flow left ventricular assisted devices (CF-LVAD), prolonging survival in end-stage heart failure patients can be seen in a new light. We also anticipate its use as a definitive therapy to overcome the limited donor organ resources for cardiac transplant. However, LVADs also have undesirable device-related complications and questionable improvement in the quality of life. In this review, we searched published articles using PubMed and Google Scholar to identify the complications and outcome of post-LVAD patients from 2014 to 2019. The studies we used included all study design types and a wide range of demographic variables focusing on age, sex, choice of LVAD as a bridge to cardiac transplant, or definitive therapy. For patients with New York Heart Association (NYHA) Class III B or IV or heart failure with reduced ejection fraction (HFrEF) with maximal medication therapy, there is a significant increase in mean ejection fraction from 4% to 6%. For patients with drug-induced cardiac toxicity or other causes of cardiac toxicity, with no significant risk factors, the ejection fraction increased to nearly 50% within 10-25 days of LVAD usage. There is also a substantial improvement in the quality of life in this literature review comparing to the pre-LVAD stage, as long as complications are taken into account. Data is limited for making an accurate judgment on the quality of life and functional capacity of LVADs. We found that the use of LVADs is not fully cost-effective, but still less financially burdening than a cardiac transplant. Although data from worldwide is limited and restricted to studies having a range of one to two years of follow-up, we conclude that LVADs are promising in improving cardiac function and the best bridging therapy available for patients waiting on a transplant.

## Introduction and background

“Declare the past, diagnose the present and foretell the future” - Hippocrates

A 37-year-old male with end-stage cardiomyopathy was transferred from a tertiary care center to the local hospital for cardiac transplant. After completing his initial evaluation, he was eligible for cardiac transplantation. Due to advanced heart failure despite standard medications, he received mechanical cardiac support with an LVAD while waiting for the transplant donor in a long waiting list.

Questions

* What will be the complication of mechanical cardiac support?

* How will his quality of life be affected while using LVAD?

* What would be the best course of action for this patient, if there is a shortage of cardiac donors or incompatibility with the donor gene?

In modern days, diagnosing heart failure has become effortless with the help of advanced medical technology, but improvement in the quality of life and survival in heart failure patients is still an impediment [1]. The growth of the heart failure population around the world becomes challenging to not only society but also to the medical field. In the United States alone, 5.8 million of the population suffer from heart failure [1]. The total incidence of heart failure patients is 650,000 yearly, and 50% of these patients reach end-stage heart failure with reduced ejection fraction with deaths of over 300,000 per year. Despite the advances in medical and surgical therapy, heart failure is still a challenging syndrome worldwide and annual costs to manage these patients is approximately $40 billion [1]. Because of this disease’s complexity and its burdensome nature both for the patients and their families, end-stage heart failure becomes one of the most challenging diseases worldwide [1-2].

However, with the introduction of mechanical circulatory support, mainly continuous-flow left ventricular assisted device (LVAD), within the last few years, there is renewed optimism in the field of cardiology to improve heart function and extend survival in patients with end-stage heart failure. Though cardiac transplant is still the ultimate therapy for heart failure, due to the shortage of donor organ resources, cardiologists and surgeons have an eye on LVAD these days as a definitive therapy [3]. Initially, an LVAD has been used as bridging to cardiac transplant to extend the survival in end-stage heart failure patient while waiting on the transplant list. With the advancement of biomedical engineering and for the sake of more convenience and availability over time, LVAD is becoming popular for use as a device more than a bridging therapy. However, it is still controversial if LVAD can be used as definitive therapy or replace cardiac transplant as LVAD has considerable device-related complications, and unanswered questions regarding life expectancy, and improvement in the quality of life [3-6].

The purpose of this study was to evaluate the outcome and convenience regarding the usage of continuous-flow LVAD, both as definitive therapy and a bridge to cardiac transplant. We sought to assess the consequences, cardiac output performance, life expectancy, and quality of life in end-stage heart failure patients within one to two years of treatment with LVAD implantation. We also wanted to investigate the benefit of using LVAD as definitive therapy in the near future to overcome the shortage of donor resources and to make it a less burdensome disease for society [4].

## Review

In this review, we used published articles indexed in PubMed and Google Scholar to identify the complications and outcome of post-LVAD patients from 2014 to 2019. There are no ethical considerations while performing this study. In addition to the usage of a set of MeSH keywords to collect the data for this literature review, an inclusion-exclusion criterion was also applied to narrow down the searches. Inclusion criteria for this literature review only consisted of articles related to human studies, studies less than five years old, clinical trials, and literature reviews. Exclusion criteria included heart failure with normal ejection fraction. Only literature in English and full-text articles were reviewed for the sake of overcoming any potential language barrier in understanding the studies and collecting more reliable information. However, the references in select articles found to be relevant were also assessed and reviewed, even if the articles themselves were published more than five years ago. 

The studies designs were included ranging from case reports, observational studies, randomized controlled trials, to systemic reviews, and meta-analysis articles. The set of MeSH keywords we used were “left ventricular assisted device”, “ejection fraction”, “death”, and “quality of life”. We found thousands of studies using each keyword and fewer in combination with one another (Table 1). After applying the filters, we found 71 articles. Eleven related articles were found by using three keywords: “left ventricular assisted device”, “ejection fraction”, “quality of life” and 60 related articles were found by using the keywords “left ventricular assisted device” and “death”. We selectively used around 50 relevant studies to perform this review.

**Table 1:** Keywords searched in PubMed and Google Scholar databases

	Keyword	Database	Number of Results
1	left ventricular assisted devices	PubMed | Google Scholar	12,536 | 267,000
2	ejection fraction	PubMed | Google Scholar	38,131 | 1,270,000
3	quality of life	PubMed	175,718
4	left ventricular assisted devices and ejection fraction	PubMed	476
5	left ventricular assisted devices and quality of life	PubMed	401
6	left ventricular assisted devices and death	PubMed	60
7	left ventricular assisted devices and ejection fraction and quality of life	PubMed	11

Demographic variables for this research include age, sex, and choice of LVAD as a bridge to cardiac transplant or definitive therapy. We focused mainly on complications and outcome of post-LVAD patients treated for end-stage heart failure and reduced ejection fraction heart failure (patients with ejection fraction less than 25% and six minutes walking distance less than 30 m) among different ages. The outcome criteria included an increase in ejection fraction at least 3% from the baseline before the LVAD treatment, and complication criteria included any cardiovascular and device-related problems starting from five days of post-LVAD treatment. There was no age limitation set for this review. Any patient, regardless of gender, ethnicity, and location, were included in this study, as long as they met our inclusion criteria. The literature review was done using only human studies research papers. Patients suffering from heart failure with an ejection fraction of >55% were excluded from this study. All the data used in this review were collected ethically using our institutional and personal access. This literature review demonstrates the outcome and convenience in New York Heart Association (NYHA) Class III or NYHA Class IV HF patients, treated with a continuous-flow LVAD (CF-LVAD) as definitive therapy, but not limited to temporary treatment awaiting a cardiac transplant or drug-induced heart failure patients during the cardiac recovery period.

**Figure 1:**
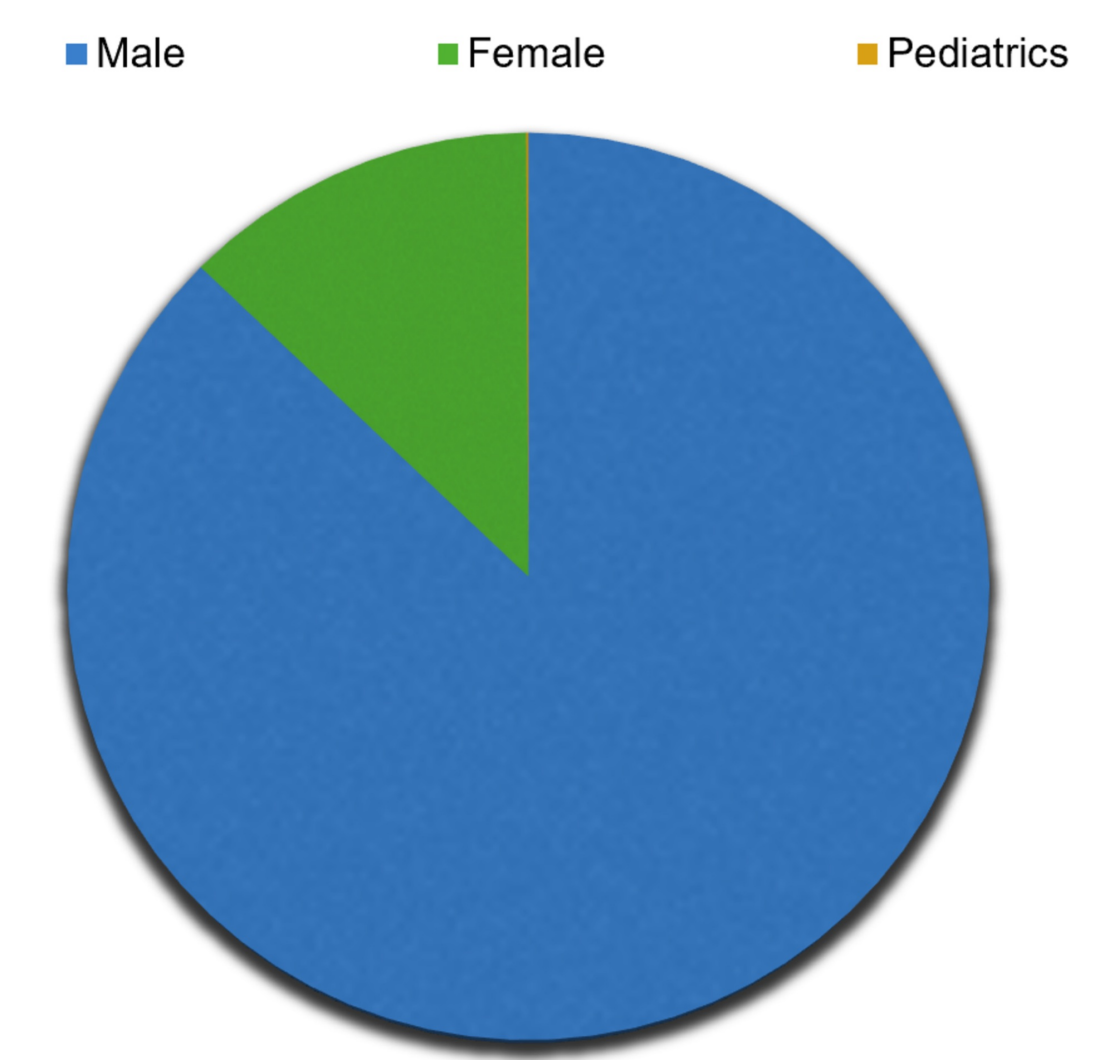
Males, females, and pediatric patients included in the review

The total number of patients in this review were 3,574 patients, with each study design possessing around 8-500 subjects, with the majority of studies having 100 patients as a whole, including both the study group and the control group. Mean age of patients was about 58 years, ranging from six to 82 years of age. Total subjects included in our review, comprised 84-85% males, 10-13% females, and 1% pediatric patients, all with either underlying ischemic, dilated, or drug-induced cardiomyopathy (Figure 1).

In this review, we focus mainly on any cardiovascular and device-related problems starting from five days of post-LVAD treatment. These included ventricular arrhythmia as the most severe adverse effect, followed by cerebral vascular accident and pump thrombosis, infections in addition to gastro-intestinal bleeding and right heart failure (including life-threatening pump failure over time). Assessing outcomes and quality of life after treatment as a bridging to transplant or definitive therapy, ejection fraction and walking distance were our main parameters. However, exercise time, peak workload, total cardiac output (TCO), peak oxygen consumption (peak VO2) and, values at the anaerobic threshold (AT) if available, were all taken into consideration. The total cost involved in LVAD treatment and maintenance of the device, along with the quality of life after LVAD treatment, was also documented.

We found that using an LVAD as definitive therapy or bridging to cardiac transplant was proven to have questionable cost-effectiveness. For patients with NYHA Class III B, NYHA Class IV, or heart failure with reduced ejection fraction (HFrEF) with maximal medication therapy, there was a significant increase in mean ejection fraction from 4% to 6% for each after LVAD treatment. The mean improvement of six minutes walking distance was from 98 m to 130 m for these patients. For patients with drug-induced cardiac toxicity or other causes of cardiac toxicity with no significant risk factors, the ejection fraction increased nearly up to 50% within 10-25 days of LVAD usage. Substantial improvement in the quality of life was observed compared to the pre-LVAD stage, with a mean increase of EQ-5D quality of life score from 19 to 28. However, the CF-LVAD is notorious for having significant morbidity and mortality due to its complications. These include drive-line infection being the most common complication, the second being an increase in bleeding risk and gastrointestinal bleeding, and third being right heart failure, hemorrhagic stroke, and pump thrombosis over time. Infrequent but fatal complications include ventricular arrhythmia within 24 hours of post-LVAD treatment and pump failure over time.

LVADs and quality of life

Evolution of LVADs

Back in 1969, the first artificial heart was invented as the treatment for end-stage heart failure patients. However, use of this first invention led to an excessive rate of hemodynamic complications, in addition to the adverse malfunction of the devices and several life-threatening outcomes. For those reasons, bio-engineering shifted its focus from total artificial heart technology to the development of LVADs. Over time, with the rapid evolution of mechanical circulatory support technology, LVAD became the promising device for end-stage heart failure patients. The first-generation volume displacement LVAD was designed to use a diaphragm and unidirectional valves to duplicate a pulsatile cardiac cycle, with diastolic filling and systolic emptying of the device. This device was approved in 2002 by the Food and Drug Administration (FDA). However, it did not become the favored choice of patients and cardiologists because of its giant pump size, adverse outcomes and limited durability (with pump failure overtime after 18-30 months of usage) [7].

**Figure 2:**
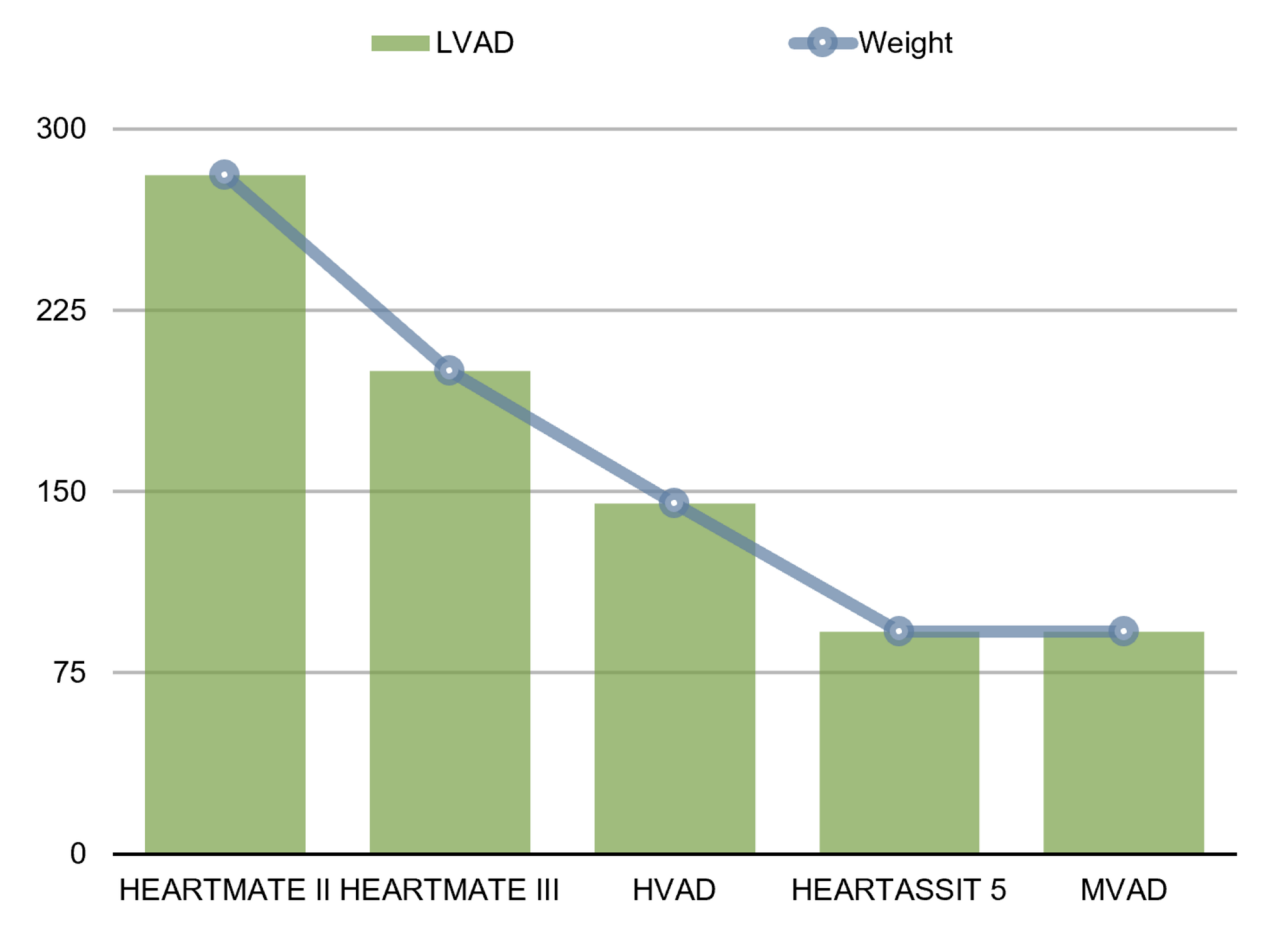
Weight (in grams) of different LVADs LVADs:  left ventricular assisted device

Within the past decade, the technology of LVAD has improved in terms of durability and smaller pump size, and LVAD has also been widely used as a bridge to transplant (BTT) and destination therapy (DT). The second- and third-generation LVADs are smaller and valve-less pumps that use a permanent magnetic field designed to rapidly spin a single impeller supported by mechanical, hydrodynamic, or magnetic bearings. Three conventional designs of new generation LVADs include axial pump LVADs, centrifugal pump LVADs, and mixed design pump LVADs [1, 8-9] (Table 2). The reduction of pump size in newer generation LVADs also helps in improving patients’ quality of life. Third-generation centrifugal pumps such as HeartMate III (Abbott Laboratories, Chicago, IL) and HeartWare ventricular assist devices, or HVAD (HeartWare Inc., Framingham, MA), have become more compact than second-generation axial pumps (e.g., HeartMate II [Abbott, Chicago, IL]). The most recent pumps, such as miniaturized left ventricular assist device, or MVAD (HeartWare Inc., Framingham, MA) and HeartAssist 5 (ReliantHeart Inc., Houston, TX), also have the lightest weight, as compared to all the other LVADs (Figure 2).

**Table 2:** Comparison of multiple LVADs

DEVICE	DESIGN	BEARING TYPE	PULSATILITY	WEIGHT(G)	MAXIMAL FLOW (L/MIN)
HEARTMATE II	AXIAL	MECHANICAL	NO	281	10
HEARTASSIST 5	AXIAL	MECHANICAL	NO	92	10
HVAD	CENTRIFUGAL	HYDRODYNAMIC	NO	145	10
HEARTMATE III	CENTRIFUGAL	MAGNETIC	YES	200	10
MVAD	MIXED	HYDRODYNAMIC	YES	92	6.5

Physiology and Complications of LVADs

The physiology of CF-LVAD reduces the arterial pulse pressure and pulsatility index to a certain extent. Because of this function, LVADs are notorious with significant morbidity and mortality, including ischemic and hemorrhagic stroke, vascular dysfunction, development of arteriovenous malformations - especially in the gastrointestinal tract, leading to GI bleeding and increased aortic stiffness. Even the formation of a small thrombus leads to disruptions to the magnetic field in the CF-LVAD, leading to sudden pump failure. According to literature and recent clinical trials, the new generation CF-LVADs (HeartMate III and HVAD) use centrifugal flow and are less prone to pump thrombosis [1, 10].

Effect on Quality of Life

Re-hospitalization plays a massive impact on the quality of life of patients with LVADs, and nearly 50-60% causes of re-hospitalization are due to device-related complications. Re-hospitalization and treatment cost due to complications can lead to poor cost-effectiveness and increased family burden with reduced quality of life for the patients. The smaller size and transcutaneously rechargeable, as well as fully implantable, devices are always a preferred option by patients. Future technology should focus more on newer LVADs with lesser complications and more efficient recharging, ultimately leading to a better quality of life of patients [1, 8-13].

Currently approved CF-LVAD devices are superior to traditional pulsatile LVADs with better-reported survival. The weight and bulk of associated batteries and controllers, along with the durability of LVADs, also have an impact on the quality of life. Future technology should focus not only on lowering the device-related complications but also on the appendages of the device and the device itself (e.g., improvement in ejection fraction with enhancement in device performance) to refine the long-term survival and quality of life for both patients and caregivers by facilitating self-care and independence.

Focus on Outcome and Cost-effectiveness

In patients suffering from end-stage HFrEF, LVADs offer new hope in terms of cardiac function and quality of life, but not as much when compared with a cardiac transplant. Because of an increasing number of cardiac transplants’ waiting lists and organ donor shortage, LVADs have become increasingly popular. The patients have to currently decide on whether they would like to wait a tremendous amount of time for a cardiac transplant or get an LVAD which, despite being a relatively quicker option, comes with a compromise in quality of life.

Ejection Fraction

According to the data collected for our review, usage of LVADs can improve quality of life around 13-15%. This was calculated based upon the EQ-5D scoring system, the improvement in ejection fraction (mean: 6%) and six-minute walking distance (mean: 20 m) in the one-to-two year period of post-LVAD studies (Table 3) [1, 8-13].

**Table 3:** Comparison of net improvement in ejection fraction after LVAD therapy LVAD: left ventricular assisted device

	Improvement of EF after LVAD Treatment	Follow-up Period	Related Articles in Reference Number	Year of Study	p-value
1	5%	3-6 months	[14]	2013	<0.001
	>10%	1 year	[14]	2013	<0.001
2	6%	3 months	[15]	2001	<0.001
	13%	6 months	[15]	2001	<0.001
3	34%	1 month	[16]	2018	<0.001
4	45%	1 month	[17]	2017	<0.001
5	52%	2 months to 2 years	[18]	2006	<0.001

**Figure 3:**
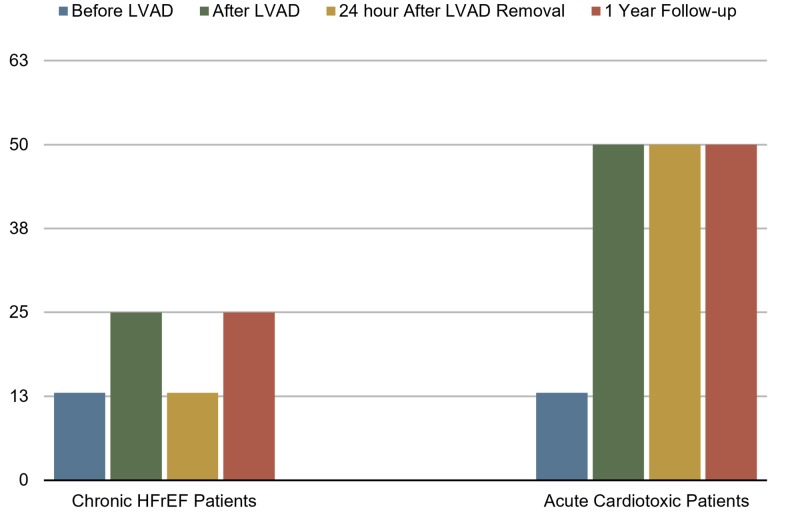
Comparison of ejection fractions before and after LVAD implantation in chronic heart failure and acute cardio-toxic patients LVAD:  left ventricular assisted device

We discovered that there is a significant improvement of ejection fraction (stroke volume/cardiac output) within one year of LVAD usage [14]. However, we can set the flow volume from the device after taking multiple parameters into consideration, including individual baseline hemodynamic parameters (invasive blood pressure, cardiac output, stroke volume, systemic vascular resistance, pulmonary vascular resistance, pulmonary artery pressure, pulmonary capillary wedge pressure, left ventricular stroke work, right ventricular stroke work, mixed venous oxygen saturation, and central venous pressure), and comorbidities such as ischemic heart disease, prolonged pulmonary hypertension, pulmonary vascular resistance, and peripheral vascular resistance [15-19]. There is an increase in mean ejection fraction by 7% with traditional HFrEF patients with NYHA Class III and NYHA Class IV, which with the help of medication, can be improved to nearly normal stroke volume. Nevertheless, in patients with temporary deterioration of cardiac function (e.g., patients with HFrEF due to cytotoxic drugs or fulminant myocarditis with normal baseline hemodynamic parameters and without co-morbidities), ejection fraction can be increased up to 50% within days of LVAD therapy. Therefore, an outcome related to ejection fraction is mainly dependent on patients’ baseline hemodynamic parameters, conjunction treatment with medication and comorbidities (Figure 3). In summary, LVAD is an excellent choice in patients waiting for cardiac recovery time. Regarding traditional HFrEF patients who are our main focus in this review, ejection fraction improvement after LVAD treatment alone does not have much impact on outcome and has a questionable improvement in patients’ quality of life [20].

Quality of Life Scoring Systems

EQ-5D scoring system used in this review is the standard scoring system for assessing the quality of life. This scoring system encompasses mobility, self-care, usual activities, pain/discomfort, and anxiety/depression, but is not limited to different scoring systems including EQ-5D-3L, KCCQ, EQ-5D-Y, EQ-5D-5L and visual analog scale (EQ-VAS). In patients using LVAD treatment as BTT or DT, their quality of life can be improved around 13-15% according to the scoring system. However, we concluded that there are also other factors, apart from improvement in ejection fraction and six minutes walking distance, that affect the quality of life score [21-22] (Table 4).

**Table 4:** Comparison of net improvement in quality of life scores

	Improvement in Quality of Life Score	Related Articles in Reference Number	Year of Study
1	EQ-5D 22.5	[22]	2018
2	EQ-5D 18.4	[19]	2017
3	KCCQ 40	[23]	2012
4	EQ-5D 20 at three months	[6]	2015
	EQ-5D 25 at six months	[6]	2015

Hospitalization

Huge hospitalization costs and family burden, including the psychosocial status of patients and caregivers play a significant role in determining the quality of life improvement after LVAD treatment. In one study, improvement in the quality of life was seen when patients were sent to a safe outpatient setting or home while remaining on the transplant list with follow-up with the health care team. Patients were found to be more confident in their capability and showed improvement in their social life and emotional well-being. This resulted in a decrease in hospital costs and caregivers’ responsibilities and was more cost-effective in the long term. However, in regards to the cost-effectiveness, there was no significant data improvement between the clinical trials due to the limited duration of the studies [23]. A prolonged course of ICU management with multiple blood works before and after LVAD treatment also have an impact on costs as it usually requires the pre-assessment of basic hemodynamic parameters along with ICU stay expenses. There are also additional costs due to LVAD device implantation by the surgical team, post-LVAD operative care requiring extended ICU management with multiple consultation teams, and several follow-up tests [19-20]. Re-hospitalization due to device-related complications after LVAD implantation, has a significant impact on the quality of life, both for the patients and the caregivers. In recent clinical trials, we found that 55% of re-hospitalization rates are due to the adverse events following DT LVAD implementation. Though medications, such as heparin, prevent pump thrombosis have little impact on the quality of life, there are bleeding complications to consider (30% of all the device-related complications), as they can lead to re-hospitalization [1, 8-13].

According to the data we collected, if the characteristics of patients selected were similar, the quality of life and survival rates were similar only for the first 60 days. Both the patients and family members should be well-informed about the balance between the improvement of survival and quality of life against post-LVAD management, financial/social burden, and risk of re-hospitalization due to LVAD therapy. Providing effective patient-centered care requires the understanding of all the risks and benefits of treatment, optimized timing for LVAD treatment in selected HFrEF patients, as well as individualizing of risk-benefit profiles for each selected patient. In conclusion, a better quality of life and improvement after targeted therapy largely depends on better post-operative care and management, and also in the psychosocial status of the patients and caregivers.

Focus on complications and cost-effectiveness

The cost of heart failure care continues to increase, not only in the United States but also worldwide, placing a heavy burden on the health care system. The overall cost of HF care was $30.9 billion in 2012 and is expected to increase to $43.6 billion in 2020 and $63.7 billion in 2030. Hospitalization cost is the main culprit for increasing the health care cost in post-LVAD patients, and that is mainly due to device-related complications [1, 9]. In this literature review, we studied the clinical trials and case reports articles relating to LVAD usage and outcome. According to the data we’ve obtained, LVAD is notorious for its fatal adverse consequences, including ventricular arrhythmia as a rare but the most serious complications within 24 hours to first 60 days of post-LVAD treatment. Other complications include cardiac tamponade concerning early bleeding risk, which requires additional surgery during the post-op period of LVAD, which may require additional surgery, device-related infections, GI bleeding, and right heart failure over time. These are followed by life-threatening pump failure, and cerebrovascular accidents are the most common device-related outcomes within one to two years of targeted therapy [1, 8-13] (Table 5).

**Table 5:** Related Complications of LVADs LVAD:  left ventricular assisted device

1	Early Bleeding (Cardiac Tamponade)	16 - 20%	within days after LVAD usage
2	Device Related Infections	16 - 24 %	within 1-2 year after LVAD usage
3	GI Bleeding	19 - 20 %	within 1-2 year after LVAD usage
4	Ischemic Stroke	16%	within 1-2 year after LVAD usage
5	Right Heart Failure	14%	within 1-2 year after LVAD
6	Haemorrhagic Stroke	8%	within 1-2 year after LVAD
7	Thrombosis	2%	within 1-2 year after LVAD
8	Arrhythmia	<1%	within 24 hours after LVAD
9	Pump Failure	2 - 3 %	within 1-2 year after LVAD

The occurrence of these complications is an important concern because of their impact on the need for re-hospitalization and the considerable increase in the cost of care. If we can control or reduce the mortality rate of these device-related complications, usage of LVAD will be more promising in the future and will give a new light to HFrEF patients as a definitive treatment. Although in this present time, LVAD has the upper hand compared to cardiac transplant in availability according to currently available statistically significant studies. However, the data we have is only for one to two years of studying LVAD usage as a bridging therapy to cardiac transplant. Thus, the usage of LVAD as a definitive treatment for HFrEF is still controversial. Nevertheless, in the future, with the advancement of technology, we hope to see new inventions of more advanced LVADs with lower complications and with more reliability and safety ratings to the currently available devices [9-24].

In summary, this review, on the advanced stage of HfrEF patients who use LVADs, focused mainly on CF-LVADs over one to two years of statistically significant studies of clinical trials and case reports. It is proven that LVADs can give overall improvement with cardiac performance. However, we should be aware of the possible adverse effects related to LVADs, as described above. Quality of life is improved compared to the pre-LVAD stage, with the overall EQ-5D score of 15% to 20%. There is also an increase in walking distance. With the invention of newer LVADs, with the battery implanted in subcutaneous tissue, leisure activities like swimming and bathing are not restricted. However, we should remember that LVADs have several unfavorable outcomes related to improper function and failure of the LVAD, which can lead to an increase in re-hospitalization - ultimately, increasing the financial burden on the family. Therefore, the cost-effectiveness of LVADs is not favorable unless we can find solutions and implement them in the newer generation of LVADs [25-26].

We deduce that the usage of CF-LVADs is a unique approach to ventricular support therapy. It is evident that CF-LVAD treatment is promising for end-stage HFrEF patients as a bridging therapy to cardiac transplant or possibly as a definitive treatment of heart failure. The outcome and convenience of post-LVAD treatment patients with prior NYHA stage III and IV heart failure patients were reviewed, and within one year of LVAD implant, many patients showed improvement in both the cardiac function and in quality of life. There is an increasing need for development and research for the newer generation of LVADs, so we may foresee using LVADs as an ultimate treatment. This is important to overcome the limited donor resources available, including very few cardiac transplant centers and the growing size of transplant waiting lists. 

Limitations

In this review, we encountered several limitations. Firstly, there is not enough published on this topic; hence, we had to also use studies that were also more than ten years old. Although our criteria include studies from all over the globe, the clinical trials we found are studies mainly originating from Europe and the United States. There are not enough published papers regarding CF-LVADs from Asia, which may be due to socioeconomic variations. Secondly, the data available is limited, as the published studies only include one to two years of follow-up of LVAD-implanted patients. Lastly, there were a few paywalls when reviewing articles, and as junior doctors, we could not access a few articles due to lack of institutional accesses to Embase and Web of Science, and our economic restrictions.

## Conclusions

CF-LVAD implants proved to improve the cardiac function and quality of life of many patients. Though ejection fraction and hemodynamic stability cannot be that of a normal healthy heart after a cardiac transplant, there is a significant improvement in ejection fraction (10% to 20%) and quality of life compared to pre-LVAD HFrEF patients. Cost-effectiveness of LVADs implantation remains unclear, but it is still less financially burdening than a cardiac transplant. It is necessary to conduct further studies on HFrEF patients, preferably from Asia, Africa, and Australia, so that we can get a broader perspective of LVAD usage worldwide. Future studies should ideally include LVAD follow-up studies with at least five years of follow-up to get more reliable data that can help us interpret whether LVAD can be used as a definitive treatment or not. For now, we conclude that using LVADs is the best short bridging therapy available for patients waiting for a transplant.
